# Meningococcal Serogroup B Bivalent rLP2086 Vaccine Elicits Broad and Robust Serum Bactericidal Responses in Healthy Adolescents

**DOI:** 10.1093/jpids/piv039

**Published:** 2015-08-04

**Authors:** Timo Vesikari, Lars Østergaard, Javier Diez-Domingo, Jacek Wysocki, Carl-Erik Flodmark, Johannes Beeslaar, Joseph Eiden, Qin Jiang, Kathrin U. Jansen, Thomas R. Jones, Shannon L. Harris, Robert E. O'Neill, Laura J. York, Graham Crowther, John L. Perez

**Affiliations:** 1 University of Tampere Medical School, Finland; 2 Department of Infectious Diseases, Aarhus University Hospital, Denmark; 3 Área de Investigación en Vacunas, FISABIO-Public Health, Universidad Católica de Valencia, Spain; 4 Department of Preventive Medicine, Poznań University of Medical Sciences, Poland; 5 Vaccine Unit, Department of Pediatrics, Skåne University Hospital, Malmo, Sweden; 6 Pfizer Ltd, Walton Oaks, Tadworth, United Kingdom; 7 Pfizer Vaccine Research, Pearl River, New York; 8 Pfizer Global Vaccines, Collegeville, Pennsylvania; 9 Pfizer Medical and Scientific Affairs, Collegeville, Pennsylvania

**Keywords:** bivalent rLP2086, clinical trial, functional immunogenicity, *Neisseria meningitidis* serogroup B, safety

## Abstract

**Background:**

*Neisseria meningitidis* serogroup B (MnB) is a leading cause of invasive meningococcal disease in adolescents and young adults. A recombinant factor H binding protein (fHBP) vaccine (Trumenba^®^; bivalent rLP2086) was recently approved in the United States in individuals aged 10–25 years. Immunogenicity and safety of 2- or 3-dose schedules of bivalent rLP2086 were assessed in adolescents.

**Methods:**

Healthy adolescents (11 to <19 years) were randomized to 1 of 5 bivalent rLP2086 dosing regimens (0,1,6-month; 0,2,6-month; 0,2-month; 0,4-month; 0,6-month). Immunogenicity was assessed by serum bactericidal antibody assay using human complement (hSBA). Safety assessments included local and systemic reactions and adverse events.

**Results:**

Bivalent rLP2086 was immunogenic when administered as 2 or 3 doses; the most robust hSBA responses occurred with 3 doses. The proportion of subjects with hSBA titers ≥1:8 after 3 doses ranged from 91.7% to 95.0%, 98.9% to 99.4%, 88.4% to 89.0%, and 86.1% to 88.5% for MnB test strains expressing vaccine­-heterologous fHBP variants A22, A56, B24, and B44, respectively. After 2 doses, responses ranged from 90.8% to 93.5%, 98.4% to 100%, 69.1% to 81.1%, and 70.1% to 77.5%. Geometric mean titers (GMTs) were highest among subjects receiving 3 doses and similar between the 2- and 3-dose regimens. After 2 doses, GMTs trended numerically higher among subjects with longer intervals between the first and second dose (6 months vs 2 and 4 months). Bivalent rLP2086 was well tolerated.

**Conclusions:**

Bivalent rLP2086 was immunogenic and well tolerated when administered in 2 or 3 doses. Three doses yielded the most robust hSBA response rates against MnB strains expressing vaccine-heterologous subfamily B fHBPs.

*Neisseria meningitidis* serogroup B (MnB) causes most meningococcal disease in Europe and approximately one third of cases in the United States [1, 2]. MnB infection is more common among adolescents and young adults than the general adult population [2]. MnB polysaccharide vaccines are poorly immunogenic, likely due to cross-reacting epitopes of MnB capsular polysaccharide with polysialic acid structures on human neuronal cells [3]. Vaccines produced from MnB outer membrane vesicles have shown clinical efficacy in children older than 4 years, against MnB strains bearing antigens homologous to those in the vaccine, but not against strains expressing antigens heterologous to the vaccine [4–6]. Therefore, alternative approaches based on conserved MnB protein antigens have been pursued to provide protection against diverse strains causing endemic and epidemic invasive MnB disease. A recombinant, multicomponent MnB vaccine (Bexsero, Novartis Vaccines, Siena, Italy) has been licensed in the European Union, Canada, Australia [7–9], and, more recently, in the United States [10]. Another multicomponent vaccine candidate, bivalent rLP2086 (Trumenba^®^), was recently approved in the United States to prevent invasive meningococcal disease caused by MnB in individuals 10 to 25 years of age [11]. The target antigen for this vaccine, LP2086, was identified using a combined biochemical and functional immunologic screening approach [12].

LP2086 is a bacterial, surface-exposed virulence factor that binds human factor H, also known as factor H binding protein (fHBP) [13]. In a comprehensive survey of 1837 MnB strains isolated from patients with invasive disease in the United States and Europe, the LP2086 gene was present in all isolates [14], although in rare cases, invasive disease-causing MnB strains may lack a functional LP2086 gene [15]. LP2086 protein sequences can be grouped into 2 genetically and immunologically distinct subfamilies, designated A (30% of isolates) and B (70% of isolates) [14]. The bivalent vaccine contains recombinant lipidated proteins from each LP2086 subfamily (bivalent rLP2086), because this formulation was shown to be most effective at inducing broadly protective serum bactericidal antibodies. In phase 1 and 2 studies, immune sera elicited by bivalent rLP2086 demonstrated bacterial killing activity, measured in serum bactericidal assays using human complement (hSBAs), against diverse MnB strains bearing fHBP variants of both subfamilies and heterologous in sequence to those in the vaccine [16–19]. For immunogenicity evaluations, 4 MnB test strains were selected to reflect the epidemiology, breadth, and global distribution of MnB strains. Each hSBA test strain expresses an fHBP variant that is different from vaccine components, thus allowing an objective assessment of functional and protective bactericidal immune responses against invasive disease-associated strains in circulation [20].

This study evaluated the immunogenicity, tolerability, and safety of bivalent rLP2086 administered in 3-dose or 2-dose regimens in healthy adolescents aged 11 to <19 years at enrollment. The immune response measured by hSBA was evaluated 1 month after dose 2 or 3 and included the percentage of subjects achieving hSBA titers ≥1:8, a more conservative indicator of potential seroprotection than the recognized correlate of protection, an hSBA titer ≥1:4 [21, 22]. Safety assessments included the incidence of local reactions, systemic events, and adverse events (AEs).

## METHODS

### Study Participants and Design

This phase 2, multicenter, randomized, single-blind study was conducted between March 3, 2011 and August 30, 2013, in healthy adolescents 11 to <19 years of age from the Czech Republic, Denmark, Finland, Germany, Poland, Spain, and Sweden. Subjects were judged healthy by the investigator and agreed to practice an effective form of contraception. Key exclusion criteria were previous vaccination with any MnB vaccine; previous anaphylactic reaction to any vaccine or vaccine-related component; history of culture-proven *N meningitidis* or *Neisseria gonorrhoeae* disease; pregnancy or nursing; current chronic use of systemic antibiotics; bleeding diathesis or condition that would contraindicate intramuscular injection; known or suspected disease of the immune system or receipt of immunosuppressive therapy; any neuroinflammatory or autoimmune condition; and receipt of any blood products, including immunoglobulin, within 6 months before the first study vaccination.

**Figure 1.**
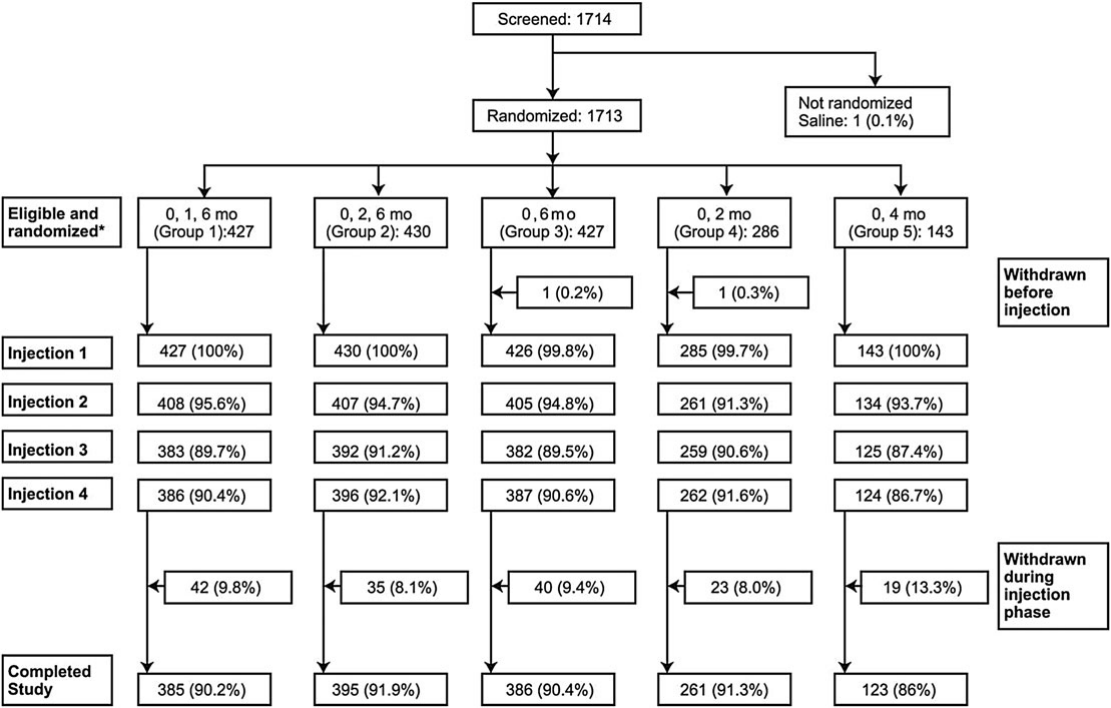
Subject disposition. *Values in this row used as denominators for percentages.

Subjects were randomized 3:3:3:2:1 corresponding to a 0,1,6-month, 0,2,6-month, 0,6-month, 0,2-month, and 0,4-month bivalent rLP2086 dosing schedule, respectively (groups 1–5) (Table 1; Figure 1). The unequal randomization schedule reflects the comparisons of interest for this study. The first 3 groups (randomized 3:3:3) were assigned to meet the hypothesis testing objectives; the remaining 2 groups (randomized 2:1) were descriptive and were used to explore different rLP2086 dosing schedules. Subjects were randomized using an interactive voice- or web-based response system. Enrollment was stratified by age (11 to <14 and 14 to <19 years) to ensure that all ages were adequately represented in the study. Each subject received 4 study injections, with administration of 2 or 3 doses of bivalent rLP2086 according to the aforementioned dosing schedule; saline was administered when bivalent rLP2086 was not scheduled. Participants were blinded to their assigned dose regimen. All laboratory staff were blinded to subject, visit, and treatment (ie, randomization group), but investigators and sponsor knew the allocation of subjects throughout the study. This study was conducted in accordance with the Declaration of Helsinki [23] and the International Conference on Harmonisation Guidelines for Good Clinical Practice [24] (ClinicalTrials.gov: NCT01299480).

**Table 1.** Study Design

rLP2086, mo	Visit 1* (Month 0)	Visit 2 (Month 1)	Visit 3* (Month 2)	Visit 4* (Month 3)	Visit 5 (Month 6)	Visit 6* (Month 7)	Visit 7 (Month 12)
	Injection 1	Injection 2	Injection 3	Blood Draw Only	Injection 4	Blood Draw Only	Telephone Contact Only
0,1,6	rLP2086	rLP2086	Saline		rLP2086		
0,2,6	rLP2086	Saline	rLP2086		rLP2086		
0,2	rLP2086	Saline	rLP2086		Saline		
0,4	Saline	Saline	rLP2086		rLP2086		
0,6	rLP2086	Saline	Saline		rLP2086		

Abbreviations: fHBP, factor H binding protein; hSBA, serum bactericidal assay using human complement; rLP2086, bivalent rLP2086 vaccine.

*Blood draw at visits 1, 3, 4, and 6 for hSBA performed with strains expressing fHBP variants A22, A56, B24, and B44.

### Vaccine and Placebo

Subjects received 1 intramuscular dose of bivalent rLP2086 or saline in the upper deltoid muscle. Each 0.5-mL dose of bivalent rLP2086 contained 60 µg each of rLP2086 A (A05) and B (B01) subfamily proteins produced in *Escherichia coli* and formulated with aluminum phosphate in an isotonic buffer in prefilled syringes. Saline (0.9% sodium chloride) was administered as a 0.5-mL dose.

### Immunogenicity Assessments

Blood samples for hSBA were obtained at 0, 2, 3, and 7 months (Table 1). Functional bactericidal antibodies were assessed against 4 MnB test strains expressing fHBP variants that are heterologous in sequence compared with the vaccine components; 2 strains express fHBP subfamily A variants (A22 and A56), and 2 strains express fHBP subfamily B variants (B24 and B44). The fHBP variants represent 4 of the 6 major fHBP variant subgroups that account for >90% of invasive meningococcal disease isolates circulating in the United States and Europe combined [20]. As an indication of the diversity of these 4 MnB test strains, the multilocus sequence type clonal complex (ie, the genetic background) of each strain represents 1 of the 4 most prevalent clonal complexes in circulating invasive MnB strains (Table 2) [20]. Each of these 4 MnB test strains have detectable fHBP surface expression levels representative of their respective fHBP variant group, as measured in a flow cytometry assay adapted from McNeil et al [25] using the anti-fHBP broadly cross-reactive monoclonal antibody MN86-994-11-1.

**Table 2.** Genotypic Characteristics of *N meningitidis* serogroup B Test Strains

Strain ID	fHBP Variant (fHBP Peptide ID^a^)	Homology,^b^ %	fHBP Subgroup	ST Clonal Complex	PorA Subtype
PMB2001	A56 (187)	98.1	N1C2	cc213	P1.22,14
PMB2707	B44 (15)	91.6	N4/N5	cc269	P1.19-1,10-4
PMB80	A22 (19)	88.9	N2C2	cc41/44	P1.21,16
PMB2948	B24 (1)	86.2	N6	cc32	P1.12-1,13-1

Abbreviations: fHBP, factor H binding protein; ID, identification; ST, sequence type.

^a^fHBP peptide ID is from http://pubmlst.org/neisseria/fHbp/.

^b^Amino acid identity to the fHBP subfamily matched vaccine component: A05 for the strains expressing fHBP subfamily A variants, or B01 for the strains expressing fHBP subfamily B variants. Within fHBP subfamily, the percentage amino acid identity is >83%.

The primary immunogenicity endpoints were the proportion of subjects receiving 3 doses of bivalent rLP2086 (vaccination at 0, 1, 6 and 0, 2, 6 months) who achieved hSBA titer ≥1:8 for each of the 4 MnB test strains 1 month after the third dose of bivalent rLP2086. An hSBA titer ≥1:8 is a more conservative indicator of seroprotection than a titer ≥1:4, which is the recognized correlate of protection against meningococcal disease [21, 22] and addresses the ability of the hSBA to determine precisely and accurately a positive from a negative seroresponse. The hypothesis-testing secondary endpoint was the proportion of subjects receiving 2 doses of bivalent rLP2086 at 0,6 months who achieved hSBA titer ≥1:8 for each of the 4 MnB test strains 1 month after the second dose of bivalent rLP2086. Other secondary immunogenicity endpoints included assessment of geometric mean titers (GMTs) for each of the 4 MnB test strains and the proportion of responders with hSBA titers ≥1:8 for all groups at each sampling point. Responses after 1 dose of bivalent rLP2086 were only available for the 0,4-month dosing group.

### Safety Assessments

Subjects were observed for ≥20 minutes (or longer per local practice) after vaccination for immediate reactions, which were documented as AEs. Reactogenicity data were collected by electronic diary (e-diary) for 7 days after each injection and included solicited local reactions and systemic events and use of antipyretic medications. Injection-site redness and swelling were measured by subjects using calipers provided, and measurements were subsequently graded as mild, moderate, or severe as defined prospectively by study protocol. Injection-site pain and systemic symptoms of headache, fatigue, chills, vomiting, diarrhea, muscle pain, and joint pain were graded by the subjects as mild, moderate, or severe. Temperature was measured at bedtime; fever was defined as temperature ≥38.0°C (100.4°F). Only the highest fever measurement recorded among measurements taken during any single 24-hour period was reported. Unsolicited AEs were collected from signing of informed consent through 1 month after the last injection (bivalent rLP2086 or saline). Serious AEs (SAEs) were collected throughout the study.

### Statistical Analysis

The evaluable immunogenicity population included subjects who received all doses of bivalent rLP2086 as randomized, had no major protocol violation or use of prohibited vaccines, had blood drawn before dose 1 and 1 month after the last dose of bivalent rLP2086, and had a valid and determinate assay result for the proposed analysis. The safety population included all participants who received at least 1 injection (bivalent rLP2086 or saline) and had any safety data available.

Immunogenicity endpoints, defined as the proportion of subjects responding with an hSBA titer ≥1:8, were summarized along with the exact 2-sided 95% confidence interval ([CI] or Clopper-Pearson confidence limit) for the proportion; the exact CI for the proportion was computed using the F distribution. The response rates in subjects receiving 3 versus 2 doses of bivalent rLP2086 were compared with 50% at a significance level of 1.25%, using a 1-sided exact test based on binomial distribution. GMTs were computed with 2-sided 95% CIs.

## RESULTS

### Subjects

A total of 1714 subjects were enrolled and 1713 were randomized; 1 subject received saline without randomization and withdrew from the study (Figure 1). A total of 1712 subjects received at least 1 dose of bivalent rLP2086 or saline, and 1550 (90.5%) completed the study. Most subjects were white (99.0%) and non-Hispanic (98.5%). At the time of first injection, 63.4% of subjects were 14–18 years of age; mean age of the total population was 14.4 years (Table 3). Demographic characteristics were similar across dosing regimens.

**Table 3.** Demographics, Safety Population

	Bivalent rLP2086 Dosing Schedule					
	0,1,6 mo n = 426	0,2,6 mo n = 414	0,6 mo n = 451	0,2 mo n = 277	0,4 mo n = 144	Total N = 1712
Sex, n (%)						
Female	212 (50)	217 (52)	227 (50)	135 (49)	79 (55)	870 (51)
Male	214 (50)	197 (48)	224 (50)	142 (51)	65 (45)	842 (49)
Race, n (%)						
White	423 (99)	408 (99)	448 (99)	272 (98)	144 (100)	1695 (99)
Age at first injection, y, n (%)						
11 to <14	155 (36)	154 (37)	164 (36)	101 (37)	53 (37)	627 (37)
14 to <19	271 (64)	260 (63)	287 (64)	176 (64)	91 (63)	1085 (63)
Mean (SD)	14.4 (2.22)	14.4 (2.23)	14.4 (2.17)	14.4 (2.25)	14.3 (2.11)	14.4 (2.20)

Abbreviation: SD, standard deviation.

### Immunogenicity

**Figure 2.**
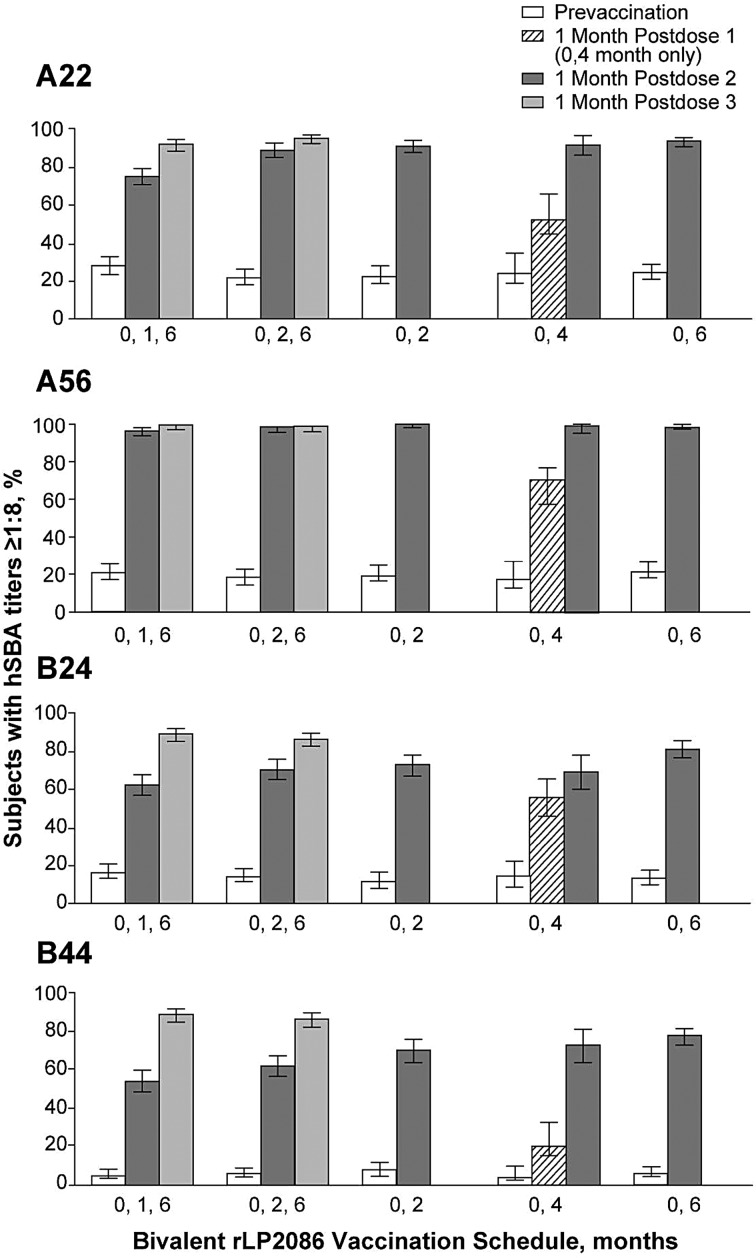
Percentage of subjects with hSBA titers ≥1:8 against *N meningitidis* serogroup B test strains A22, A56, B24, and B44 at baseline and 1 month after injection with bivalent rLP2086 or saline. Errors shown are 95% confidence intervals. hSBA, human serum bactericidal antibody assay using human complement.

A total of 1450 subjects comprised the evaluable immunogenicity population. One month after dose 3, the proportion of subjects with hSBA titers ≥1:8 after 3 doses of bivalent rLP2086 administered at 0,1,6 months was 91.7%, 99.4%, 89.0%, and 88.5% for MnB test strains expressing vaccine-heterologous fHBP variants A22, A56, B24, and B44, respectively. For subjects vaccinated at 0,2,6 months, 95.0%, 98.9%, 88.4%, and 86.1% had hSBA titers ≥1:8 (Figure 2). There were no significant differences in immunogenicity among subjects who received bivalent rLP2086 on the 0,1,6-month and 0,2,6-month schedules.

For fHBP subfamily A strains, among those receiving 2 doses of bivalent rLP2086, >90% of subjects had hSBA titers ≥1:8 for A22 and >98% of subjects had hSBA titers ≥1:8 for A56 1 month after the second dose. For fHBP subfamily B strains, >69% of subjects had hSBA titers ≥1:8 for B24 and >70% for B44 (Figure 2). The study design allowed assessment of hSBA responses at 1 month after 1 dose of bivalent rLP2086 (0,4-month group only). After the first dose, 55.9%, 67.6%, 56.9%, and 23.8% of subjects had hSBA titers ≥1:8 for A22, A56, B24, and B44, respectively. Prevaccination hSBA responses to all 4 MnB test strains were low, ranging from 5.2% (B44) to 28.1% (A22).

**Figure 3.**
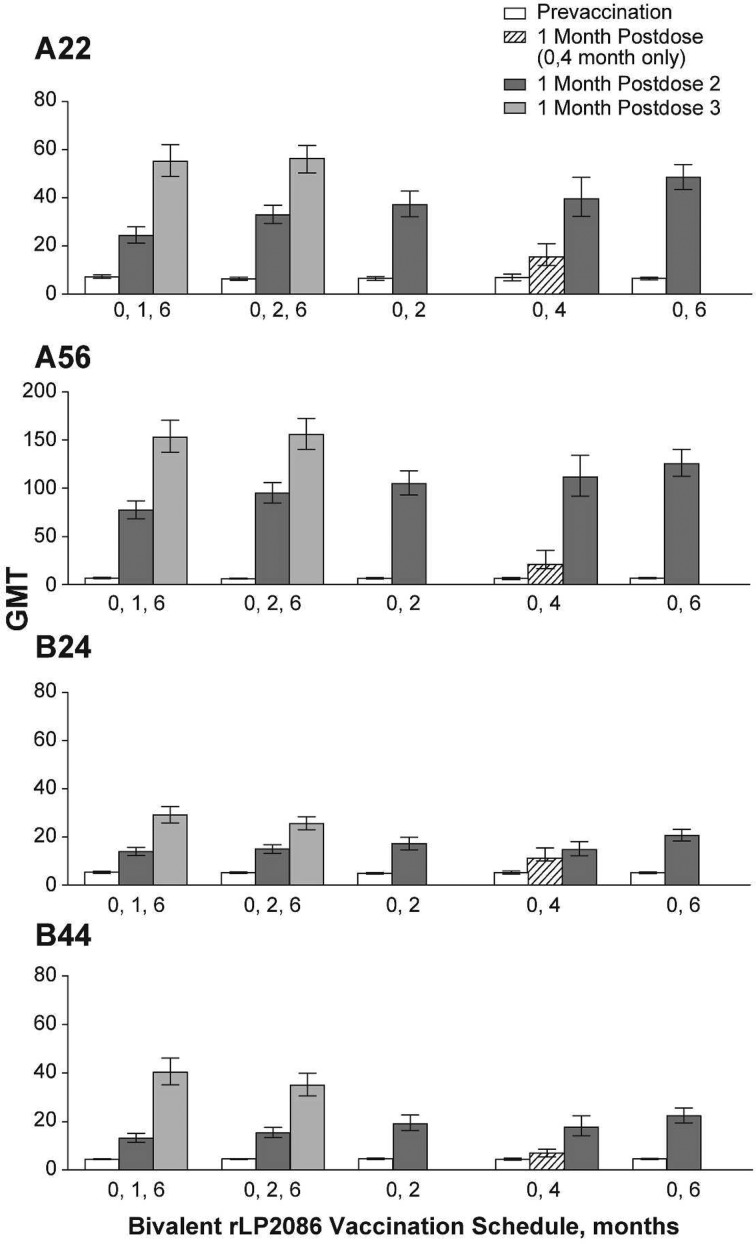
Geometric mean titers (GMTs) against *N meningitidis* serogroup B test strains A22, A56, B24, and B44 at baseline and 1 month after injection with bivalent rLP2086 or saline.

Postvaccination GMTs increased with each dose of bivalent rLP2086 and were highest among subjects receiving 3 doses of bivalent rLP2086. For subjects vaccinated at 0,1,6 months, GMTs 1 month after dose 3 were 55.1, 152.9, 29.1, and 40.3 for A22, A56, B24, and B44, respectively. For subjects vaccinated at 0,2,6 months, GMTs were 56.3, 155.6, 25.6, and 35.0 (Figure 3). For fHBP subfamily A strains, among subjects receiving 2 doses of bivalent rLP2086, GMTs ranged from 37.1 to 48.4 for A22, and 104.9 to 125.6 for A56 one month after the second dose. For the fHBP subfamily B strains, GMTs ranged from 14.7 to 20.6 for B24 and 17.8 to 22.5 for B44. After 2 doses of bivalent rLP2086, GMTs trended numerically higher among subjects with a longer interval between doses; subjects with a 6-month interval between doses (0,6-month group) had higher GMTs to all 4 MnB test strains compared with subjects having a 4-month (0,4-month), 2-month (0,2- and 0,2,6-month), or 1-month (0,1,6-month) interval between doses (Figure 3). After 1 dose of bivalent rLP2086 (0,4-month group), GMTs were 16.0, 26.8, 12.6, and 6.8, respectively, for A22, A56, B24, and B44.

### Safety

**Figure 4.**
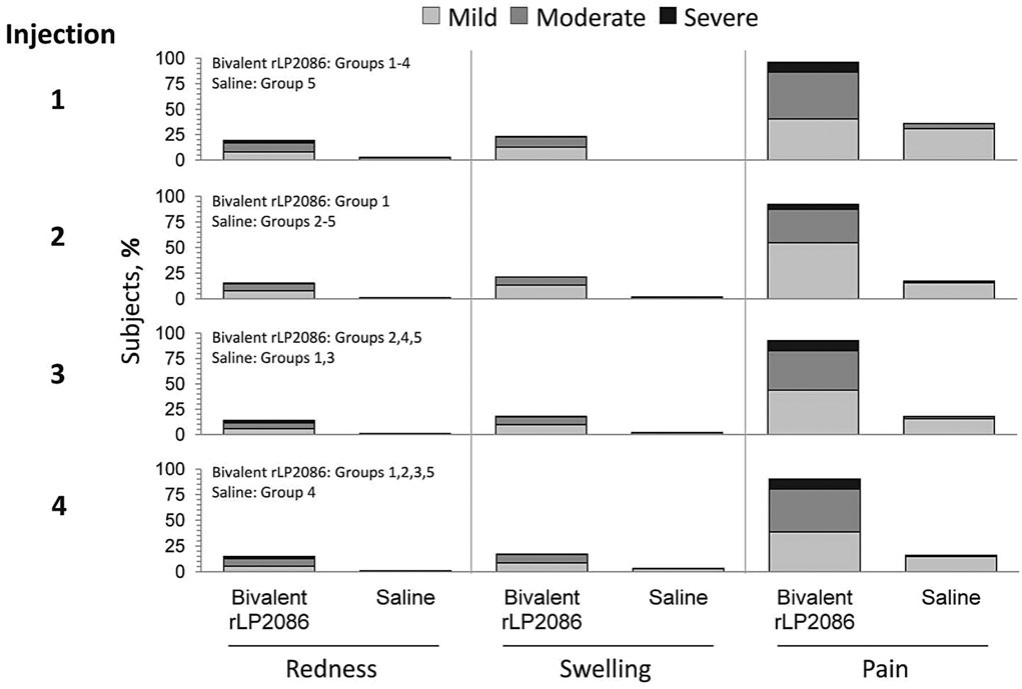
Local injection-site reactogenicity (recorded by electronic diary [e-diary]). Data have been aggregated across groups to show reactogenicity after each dose.

The frequency of local reactions was higher after bivalent rLP2086 administrations compared with saline; pain at the injection site was the most common local reaction (Figure 4). Across dosing schedules, most cases of pain after bivalent rLP2086 and saline administration were mild or moderate. Severe pain was reported by ≤9.9% of subjects who received bivalent rLP2086 and ≤0.3% subjects who received saline, for each of the 4 injections. Other common local reactions included redness and swelling, which were mild or moderate in severity (Figure 4). The mean duration of all local reactions after each dose of bivalent rLP2086 was 2.1 to 3.2 days; 4 local reactions, all of which were pain at the injection site, had a duration greater than 14 days, but no subjects withdrew from the study due to pain.

**Figure 5.**
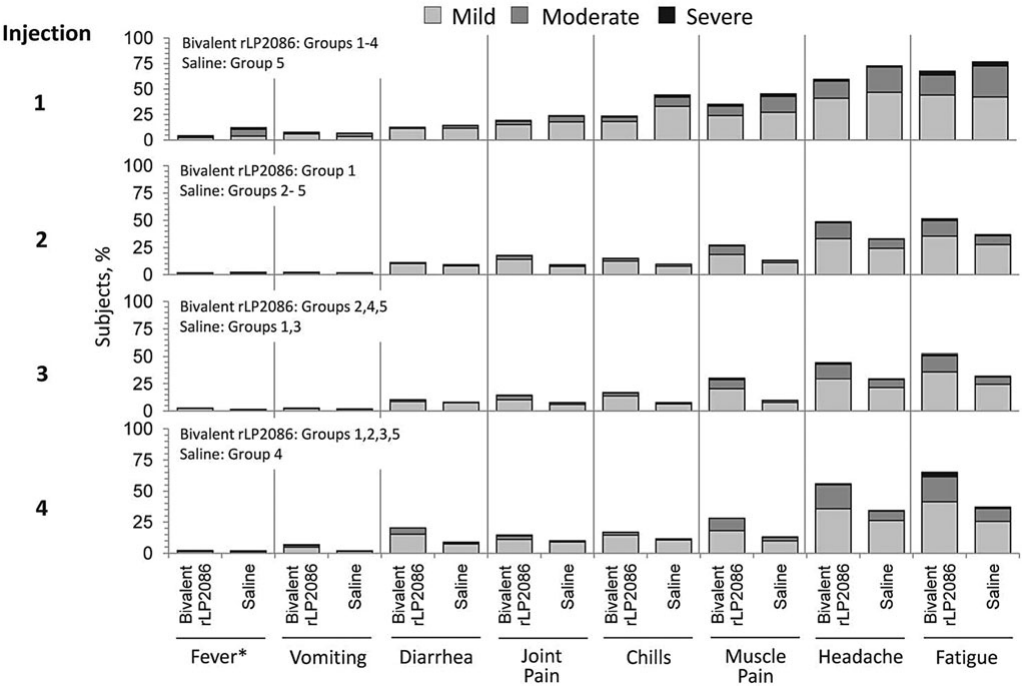
Systemic reactogenicity (recorded by electronic diary [e-diary]). Data have been aggregated across groups to show reactogenicity after each dose. *Fever: 38.0–38.4°C = mild; 38.5–38.9°C = moderate; and ≥39°C = severe.

Across all injections, the most common systemic events were headache and fatigue. The majority of systemic events were mild or moderate in severity. Severe headache was reported by ≤1.6% of subjects after either bivalent rLP2086 or saline. Severe fatigue was reported by ≤3.6% of subjects after either injection (Figure 5). In total, 3 subjects withdrew due to a systemic event.

Fever ≥38°C within 7 days of vaccination was infrequent, reported by 1.7%–4.3% and 1.5%–2.1% of bivalent rLP2086 or saline recipients, respectively. Fever ≥39°C was infrequent (<1% across groups). The median duration of fever was 1 day after bivalent rLP2086 or saline administration. Across all 4 injections, 13.6%–16.2% and 7.5%–9.1% of subjects used antipyretic medication after bivalent rLP2086 and saline, respectively.

During the vaccination phase, 35.5%–37.5% of subjects reported ≥1 AE across the 5 dosing groups. Most events were mild or moderate in severity. The most common AE was nasopharyngitis (5.5% − 10.1%). Eleven subjects experienced severe AEs considered to be related to bivalent rLP2086, including headache, injection-site pain, pyrexia, vomiting, and injection-site swelling. Two subjects reported SAEs considered to be related to bivalent rLP2086; 1 subject experienced vertigo, chills, and headache after dose 3, and another experienced pyrexia and vomiting after dose 1. Overall, there were no differences in the incidence of SAEs between bivalent rLP2086 and saline recipients or between the 3-dose and 2-dose schedules and no increase in AEs with subsequent dosing. Nineteen subjects (1.1%) withdrew due to an AE. Of these, 9 AEs were considered to be related to study vaccination and included injection-site pain (n = 4), headache (n = 2), migraine, fatigue, and vertigo (n = 1 each). No deaths were reported.

## DISCUSSION

Meningococcal B infection can lead to severe and debilitating disease. Vaccination against non-B meningococcal serogroups (ACWY) has proven efficacious in all age groups, particularly in adolescents who are at high risk for meningococcal disease [26]. The need for an effective MnB vaccine is underscored by recent outbreaks on US college campuses and by endemic infections worldwide [27–29]. The low basal immunity to meningococcal B strains in this study emphasizes the vulnerability of adolescents to infection and disease caused by *N meningitidis* serogroup B.

In this study, all 5 bivalent rLP2086 vaccination regimens, regardless of schedule or frequency, were immunogenic and well tolerated in healthy adolescents. The 2-dose and 3-dose regimens elicited robust immune responses. Considering that hSBA titers ≥1:4 are an accepted correlate for protection against invasive meningococcal disease [21, 22] and that seroprotective responses in this study were defined using more stringent criteria of hSBA titers ≥1:8, bivalent rLP2086 has demonstrated a substantial and broad immune response after 2 doses and a more robust response after 3 doses, when administered across a range of dosing schedules. This study also allowed assessment of hSBA responses after 1 dose of bivalent rLP2086. After a single vaccination, increased seroprotective responses compared with baseline against all MnB test strains were observed.

Among recipients receiving 3 doses of bivalent rLP2086, the timing of the second vaccination, whether 1 or 2 months after initial vaccination, had no discernable effect on immunogenicity. Nonetheless, the ability to elicit robust responses after 2 doses 1 month apart may be valuable during outbreaks when timely protection is critical. Immune responses after 2 doses of bivalent rLP2086 were substantial and indicative of a broad immune response to the MnB test strains that were selected to represent epidemiologically and antigenically relevant invasive MnB strains. Immunogenicity generally increased in the 2-dose schedules when there was a longer interval between the first and second dose, as seen by the higher GMTs and generally higher proportion of subjects with hSBA titers ≥1:8 after dose 2 in the 0,6-month dosing regimen compared with the other dosing schedules. Similar observations were described for quadrivalent human papillomavirus vaccination [30, 31]. In these studies, increasing the interval between doses 2 and 3 from 4 to 10 months resulted in higher antibody titers against all 4 human papillomavirus types examined.

Similar to other clinical studies of bivalent rLP2086 in adolescents and adults [18, 19, 32, 33], the majority of safety events observed in this study were local reactions and systemic events that were mild or moderate in severity, transient, and without potentiation at subsequent dosing. Approximately two thirds of subjects did not report any AEs during the study. Overall, 90.6% of subjects were able to complete their vaccination series, indicating that vaccinations were well tolerated in adolescents and that any safety events, such as injection-site pain, were not an impediment to vaccination. Safety events were not increased in subjects receiving a third dose of bivalent rLP2086.

## CONCLUSIONS

In summary, 2 or 3 doses of bivalent rLP2086 were immunogenic and well tolerated. The 2-dose regimens provided substantial hSBA responses against diverse MnB strains expressing fHBP variants heterologous to vaccine antigen. However, the 3-dose regimens yielded the highest seroconversion rates against subfamily B strains and a higher level of hSBA antibodies as measured by GMTs. The convenience of a 2-dose schedule and the potential benefit of higher level of protective antibodies after a 3-dose schedule should be carefully considered for future MnB immunization schedules.
